# Meshless Search SR-STAP for Airborne Radar Based on Meta-Heuristic Algorithms

**DOI:** 10.3390/s23239444

**Published:** 2023-11-27

**Authors:** Yunfei Hou, Yingnan Zhang, Wenzhu Gui, Di Wang, Wei Dong

**Affiliations:** The State Key Laboratory on Integrated Optoelectronics, Jilin University, Changchun 130012, China; houyf22@mails.jlu.edu.cn (Y.H.); zyn22@mails.jlu.edu.cn (Y.Z.); gwzwww@163.com (W.G.)

**Keywords:** space-time adaptive processing (STAP), off-grid effect, clutter subspace, meta-heuristic algorithms

## Abstract

The sparse recovery (SR) space-time adaptive processing (STAP) method has excellent clutter suppression performance under the condition of limited observation samples. However, when the cluttering is nonlinear in a spatial-Doppler profile, it will cause an off-grid effect and reduce the sparse recovery performance. A meshless search using a meta-heuristic algorithm (MH) can completely eliminate the off-grid effect in theory. Therefore, genetic algorithm (GA), differential evolution (DE), particle swarm optimization (PSO), and grey wolf optimization (GWO) methods are applied to SR-STAP for selecting exact clutter atoms in this paper. The simulation results show that MH-STAP can estimate the clutter subspace more accurately than the traditional algorithm; PSO-STAP and GWO-STAP showed better clutter suppression performance in four MH-STAP methods. To search for more accurate clutter atoms, PSO and GWO are combined to improve the method’s capacity for global optimization. Meanwhile, the fitness function is improved by using prior knowledge of the clutter distribution. The simulation results show that the improved PSO-GWO-STAP algorithm provides excellent clutter suppression performance, which solves the off-grid problem better than does single MH-STAP.

## 1. Introduction

Space-time adaptive processing (STAP) is an effective technique to suppress clutter received by airborne radar and enhance its ability to detect moving targets [1,2,3]. However, traditional STAP methods face challenges in acquiring sufficient training samples from uniform clutter environments, a requirement that is often difficult to fulfill in practical scenarios [4,5]. Sparse recovery (SR) approaches have demonstrated high accuracy in target signal recovery by selecting vectors from an overcomplete dictionary [6,7]. SR-STAP builds the sparse dictionary by discretizing the spatial-Doppler plane, which causes inaccuracy regarding clutter atoms not located at grid points and the degradation of clutter suppression performance [8,9]. This phenomenon is known as the off-grid problem in SR-STAP [10]. Although the off-grid effect can be weakened by reducing the mesh size and increasing the number of vectors in the overcomplete dictionary, this will also bring about problems such as an increase of computation and a strong coherence between space-time steering vectors [11].

Recent research has increasingly explored the integration of deep learning with STAP. The CNN-STAP method establishes a mapping relationship between low-resolution and high-resolution clutter angle-Doppler spectra, subsequently utilizing the CNN output to select main clutter component atoms for the reconstruction of the clutter covariance matrix (CCM) [12]. An enhancement of this approach, DU-CNN-STAP, incorporates deep unfolding (DU) concepts to refine the SR-STAP algorithm. This method leverages a high-resolution clutter angle-Doppler spectrum as input for the CNN, offering more accurate clutter space-time spectrum estimation and reducing the CNN network scale compared to the original CNN-STAP [13]. Additionally, deep learning has been applied to moving target indication (MTI) in the STAP context. This novel approach inputs a low-resolution clutter-plus-target angle-Doppler spectrum into the network, producing a high-resolution target angle-Doppler spectrum output. It demonstrates the potential to accurately predict targets with a limited number of samples [14]. In summary, deep learning-based STAP methods differ significantly from conventional SR-STAP approaches, primarily by establishing suitable mapping relationships through extensive data training. Consequently, deep learning-based STAP can be regarded as an offline process, which can be effectively combined with SR-STAP.

To solve the off-grid problem in SR-STAP, the reduced-dimension local search clutter subspace estimation STAP (RD-LSCSE-STAP) algorithm was introduced in [15]. The RD global STAP dictionary is designed to lessen computational demands, but the accuracy of the grid still depends on the grid division of the local dictionary. The local mesh splitting subspace estimation STAP (LMSSE-STAP) algorithm further enhances performance through continuous local mesh splitting [16]. However, the above methods essentially seek more accurate atoms by increasing the mesh density, which may not fully eliminate the off-grid effect.

A novel approach, the gridless STAP algorithm based on particle swarm optimization (PSO-STAP), was proposed in [17]. This algorithm employs PSO to continuously search for atoms in the spatial-Doppler profile without using a mesh dictionary, potentially eliminating the off-grid effect and accurately determining the clutter subspace. However, it lacks comparative analysis with other meta-heuristic algorithms, and its clutter suppression performance requires further enhancement.

Meta-heuristic algorithms (MH) are effective methods to solve global optimization problems, which mainly simulate natural phenomena to achieve the optimal solution. They are categorized into evolutionary computation (EC) and swarm intelligence (SI) algorithms [18]. Within EC algorithms, genetic algorithms (GA) and differential evolution (DE) are well-regarded, while particle swarm optimization (PSO) and grey wolf optimization (GWO) represent SI. At present, meta-heuristic algorithms have been successfully applied in many fields. According to [19] and [20], a meta-heuristic algorithm has worked in decentralized detection based on wireless sensor networks (WSNs), and PSO-based quantizers have optimized the performance of WSN. A dynamic modified chaotic PSO algorithm is proposed for radar signal sorting in [21], which provides stable and fast performance with excellent sorting indexes. In addition, meta-heuristic algorithms are also widely used to achieve high-accuracy estimation of direction of arrival (DOA) [22,23]. There are fewer studies on the role of meta-heuristic algorithms in solving the off-grid problem of SR-STAP directly.

In this study, several meta-heuristic algorithms (GA, DE, PSO, and GWO) are employed to address the off-grid problem in SR-STAP. These algorithms select accurate atoms by maximizing a suitable fitness function in a gridless spatial-Doppler profile. The findings indicate that meta-heuristic algorithms significantly reduce the off-grid effect in SR-STAP. Specifically, PSO and GWO, as SI algorithms, construct more precise clutter subspaces compared to other algorithms. Furthermore, building on these findings, the PSO-GWO-STAP algorithm is developed, amalgamating the strengths of PSO and GWO. Concurrently, the fitness function is improved to boost the algorithm’s performance under certain conditions. The results demonstrate that the PSO-GWO-STAP algorithm outperforms the PSO-STAP algorithm, showing its suitability for SR-STAP applications.

The rest of this paper is organized as follows. Section 2 introduces the signal model and the performance of basic MH-STAP methods. In Section 3, PSO-GWO-STAP is proposed, and the fitness function is improved according to the clutter distribution. Accordingly, performance analysis is performed in this section. Finally, the conclusion and further avenues of research are given in Section 4.

Notation: Boldface uppercase letters represent matrices, and boldface lowercase letters represent vectors. *T* and *H* represent transposition and conjugate transposition operations, respectively. The symbol ⊗ denotes the Kronecker product operation. $||•|{|}_{0}$ and $||•|{|}_{2}$ denote the *l_0_* norm and *l_2_* norm, respectively. ${\left(\cdot \right)}^{†}$ is the Moore–Penrose inverse operator.

## 2. Background and Basic Method

### 2.1. Signal Model

**Figure 1:**
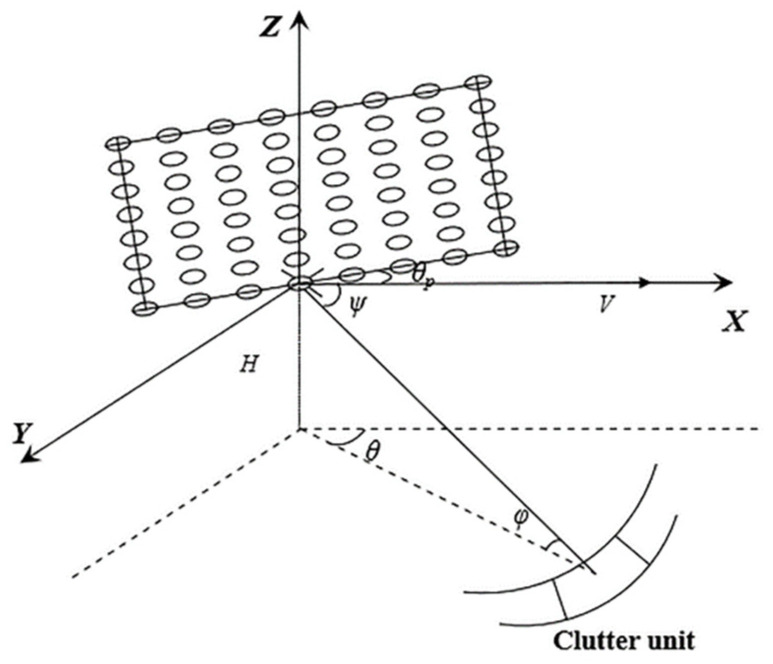
Geometric model of UPA airborne radar.

The geometric model of an airborne radar system employing a uniform planar array (UPA) consisting of *M* × *N* elements is depicted in Figure 1. Here, *θ* and *φ* denote the azimuth and pitch angles, respectively. *ψ* is defined as the cone angle, and *θ_p_* signifies the angle between the antenna plane and the direction of the flight velocity *V*. The altitude of the platform is denoted by *H*. Moreover, the radar operates at a wavelength of *λ*, with an array spacing of *d* and a pulse repeat frequency of *f_r_*, and it transmits *K* pulses in each coherent processing interval (CPI).

To streamline computational demands, the system can be equivalently represented as an N-element equally spaced linear array [24]. The space-time clutter plus noise snapshot for the *l*th range cell can be expressed as:

(1) $$X_{l}=\sum_{i=1}^{N_{c}}\gamma_{i}S(f_{d,i},f_{s,i})+N$$

(2) $$S(f_{d,i},f_{s,i})=s_{d}(f_{d,i})⊗s_{s}(f_{s,i})$$

where *N_c_* represents the number of independent clutter units and **N** refers to the white noise received by the radar. $\gamma_{i}$ is the complex amplitude. **S** is the space-time steering vector, and ⊗ denotes the Kronecker product operation. $s_{d}$ and $s_{s}$ denote temporal and spatial steering vectors, respectively, and their formulas are as follows:

(3) $$s_{d}(f_{d,i})={\left[1,\exp(j2\pi f_{d,i}),\ldots ,\exp(j2\pi (K-1)f_{d,i})\right]}^{T}$$

(4) $$s_{s}(f_{s,i})={\left[1,\exp(j2\pi f_{s,i}),\ldots ,\exp(j2\pi (N-1)f_{s,i})\right]}^{T}$$

In SR-STAP, the spatial-Doppler plane is uniformly dispersed into *N_s_ = ρ_s_ N* and *N_d_ = ρ_d_ K* grid points along the spatial and temporal axes, respectively, where *ρ_d_, ρ_s_* > 1 determine the resolution of the plane. Assuming all clutter units are precisely located on these grid points, Formula (1) can be reformulated as:

(5) $$X_{l}=\Phi \alpha +N$$

(6) $$\Phi =\left[S(f_{d,1},f_{s,1}),\ldots ,S(f_{d,1},f_{s,N_{s}}),\ldots ,S(f_{d,K_{d}},f_{s,N_{s}})\right]$$

where **Φ** is the *NK* × *N_s_N_d_* overcomplete dictionary composed of space-time steering vectors. α is the sparse coefficient, which can be solved by the following formula:

(7) $$\min||\alpha |{|}_{0},\;s.t.\;||X_{l}-\Phi \alpha |{|}_{2}^{2}\leq \sigma_{n}^{2}$$

where $||•|{|}_{0}$ and $||•|{|}_{2}$ denote the *l_0_* norm and *l_2_* norm of the vector, respectively, and $\sigma_{n}^{2}$ is the noise threshold.

### 2.2. Basic MH-STAP Method

Meta-heuristic algorithms typically commence with multiple candidate solutions as initial values. These algorithms calculate the objective function value based on these initial values and update the candidate solutions through various methods, eventually progressing to the next iteration cycle. Theoretically, meta-heuristic algorithms are expected to locate the global optimum over time. However, it is acknowledged that no single algorithm is universally effective for all problem types. Consequently, although the basic PSO-STAP method has been proposed, four meta-heuristic algorithms (PSO, GWO, DE, and GA) are employed to address the off-grid problem. Their optimization performances in relation to SR-STAP are compared in this section.

#### 2.2.1. MH-STAP Algorithm Flow

In the PSO algorithm, particles are characterized by two attributes: position and velocity. The velocity of a particle determines its position in the subsequent iteration. Particles follow the current optimal particle to search in the space [25]. GWO executes optimization through three processes, namely, tracking, encircling, and attacking prey, and it requires the setting of fewer parameters compared to PSO [26]. The differential evolution algorithm consists of three parts: mutation, crossover, and selection. It contains two critical unknown parameters: the scale factor *F* in the mutation operation and crossover rate *CR* in the crossover operation [27]. The standard genetic algorithm employs three types of genetic operators for iteration: a selection operator, crossover operator, and mutation operator. “Roulette wheel selection” is the most common method used to carry out the process of survival of the fittest among individuals. New individuals are then generated according to the crossover probability *P_c_* and mutation probability *P_m_* [28].

The fitness function is crucial for determining the optimal solution in meta-heuristic algorithms. Fewer space-time vectors are expected to be selected to estimate the clutter subspace for SR-STAP methods. Therefore, an appropriate space-time steering vector can be selected by maximizing the fitness function in each iteration. The basic fitness function formula is as follows:

(8) $$F(a)=\frac{\left|S{\left(f_{d},f_{s}\right)}^{H}P_{n}^{k}\hat{R}P_{n}^{k}S(f_{d},f_{s})\right|}{\left|S{\left(f_{d},f_{s}\right)}^{H}P_{n}^{k}S(f_{d},f_{s})\right|}$$

where $\hat{R}=\Sigma_{l=1}^{L}X_{l}X_{l}/L$ denotes a sample covariance matrix (SCM) of L samples. $a=\left[f_{d},f_{s}\right]$ represents the candidate solution, including two dimensions (spatial frequency and Doppler frequency). And *k* represents the number of external iterations. $P_{n}$ denotes a projection matrix on the noise subspace and $P_{n}^{k}=I-\Phi_{s}^{k}{\left(\Phi_{s}^{k}\right)}^{†}$. $\Phi_{s}^{k}$ represents the set of space-time steering vectors of the selected atoms, and ${\left(\cdot \right)}^{†}$ is the Moore–Penrose inverse operator. Moreover, $P_{n}^{k}\hat{R}P_{n}^{k}$ can be understood as the residual of $\hat{R}$ in the current iteration. The numerator represents the atomic response to the residual, and the denominator can be considered a normalization operation.

A previous study has established that the typical population size for PSO ranges between 20 and 50 [29]. Consequently, the population size for the four algorithms is uniformly set to 50. Subsequent testing determined the optimal number of iterations: 50 for PSO and GWO, 100 for DE, and 300 for GA. (When calculating the average runtime, the number of iterations is set to 50 for all algorithms.) Although increasing the population size and the number of iterations may enhance performance, it also significantly increases computational demands. In addition, the boundary conditions of the four algorithms are set as [−1, 1] according to the normalized space and Doppler frequency range. The input parameters of algorithms are also optimized: *P_c_* = 0.7 and *P_m_* = 0.2 for GA, and *F* = 0.5 and *CR* = 0.9 for DE. The parameters of PSO are consistent with [17]. The generalized process for MH-STAP is as follows:

(1) Initialization: *k* = 0, $P_{n}^{0}=I$, $E^{0}=\mathrm{tr}(\hat{R})/(NK)$, $\Phi_{s}^{0}=\left[\right]$.
(2) Optimization: obtain the optimal space-time steering vector $S^{k}$ under the current iteration by using meta-heuristic algorithms.
(3) Update: *k* = *k* + 1, $\Phi_{s}^{k}=\left[\Phi_{s}^{k-1},S^{k}\right]$, $P_{n}^{k}=I-\Phi_{s}^{k}{\left(\Phi_{s}^{k}\right)}^{†}$, $E^{k}=\mathrm{tr}(P_{n}^{k}\hat{R}P_{n}^{k})/(NK)$.
(4) Judgment: When the criterion $E^{k}<\eta \sigma_{n}^{2}$ is satisfied, jump out of the loop.

Then, the weight vector of STAP is calculated by:

(9) $$w=P_{n}S_{0}$$

where $S_{0}$ is the steering vector of target.

#### 2.2.2. Performance Comparison

In this letter, the performances of four algorithms applied to SR-STAP are evaluated and compared with the traditional algorithm under the condition of UPA with the parameters in the Table 1.

**Figure 2:**
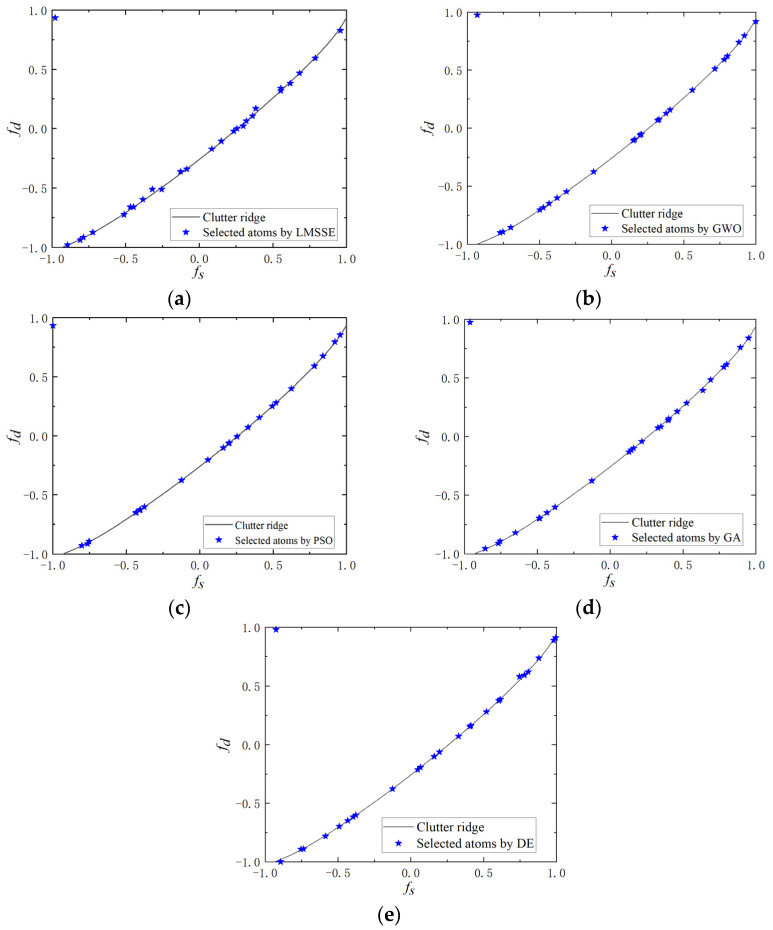
Selected atom distributions of (**a**) LMSSE-STAP, (**b**) GWO-STAP, (**c**) PSO-STAP, (**d**) GA-STAP, and (**e**) DE-STAP.

The first experiment focused on comparing the distributions of atoms in GA-STAP, DE-STAP, PSO-STAP, GWO-STAP, and LMSSE-STAP ($\rho_{d}=\rho_{s}=6$) within the spatial-Doppler profile, under the condition of *η* = 0.8. The global meshless search characteristic of the four meta-heuristic algorithms resulted in atoms being predominantly located on the actual clutter ridge, as illustrated in Figure 2. Meanwhile, the difference between the four meta-heuristic algorithms is inconspicuous. LMSSE-STAP depends on the resolution of the initial mesh and the number of local mesh splits, so the presence of atoms that deviate from the clutter ridge is difficult to avoid.

**Figure 3:**
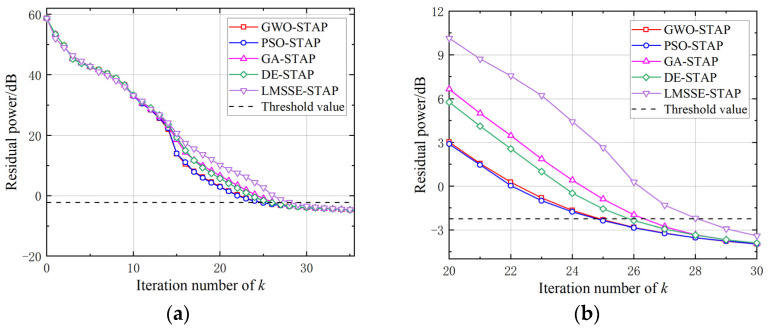
Comparison of residual power. (**a**) 0–35 iterations. (**b**) 20–30 iterations.

The second experiment aimed to compare the residual power E of the five algorithms, which is the average of 100 Monte Carlo trails (similarly hereinafter). As shown in Figure 3, LMSSE-STAP exhibited a higher residual power after the 15th iteration and required more iterations to reach the threshold.

Among the four MH-STAP algorithms, PSO and GWO exhibited the most rapid decline in residual power, followed by DE, with GA showing the slowest rate of decline. When the number of iterations exceeded 33, the residual powers of the five algorithms converged to a similar level. Reaching the threshold requires 25 iterations for PSO-STAP and GWO-STAP, 26 for DE-STAP, 27 for GA-STAP, and 29 for LMSSE-STAP. This indicates that meta-heuristic algorithms require fewer atoms to represent the clutter subspace than LMSSE-STAP.

**Figure 4:**
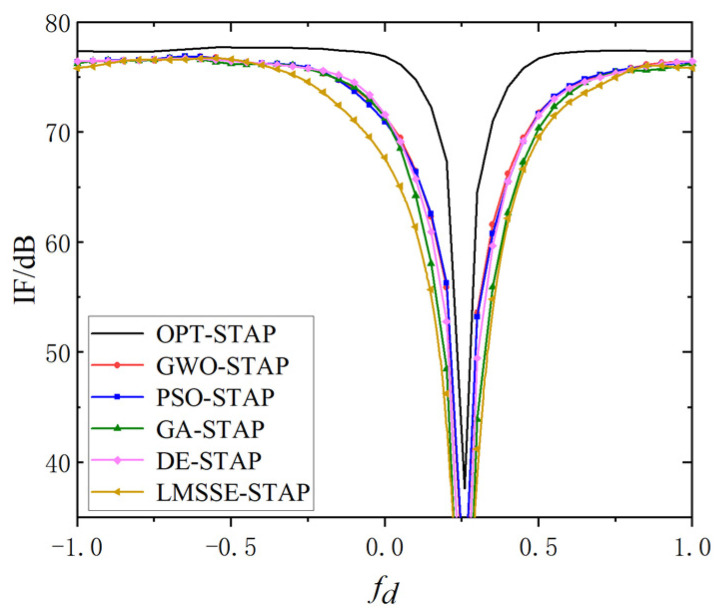
Comparison of IF curve.

The third experiment is set to evaluate the improvement factor (IF) of the five algorithms in the same situation, which is defined as:

(10) $$\mathrm{IF}=\frac{{\left|w^{H}s_{s}\right|}^{2}}{w^{H}Rw}\cdot \frac{\mathrm{tr}(R)}{s_{s}^{H}s_{s}}$$

where R denotes the exact clutter covariance matrix. IF curves of the five algorithms are shown in Figure 4, and LMSEE-STAP demonstrates the least effective performance. The four meta-heuristic algorithms continued to exhibit robust performance in this part. Swarm intelligence (SI) algorithms outperformed evolutionary computation (EC) algorithms, possibly due to their inherent nature, as clutter is regularly distributed on the spatial-Doppler plane, favoring the search capabilities of SI. While the performance of EC algorithms could be enhanced by increasing the number of iterations, this would lead to a substantial computational burden.

Finally, the computational complexities of the four kinds of STAP based on meta-heuristic algorithms are analyzed. The time complexity of MH-STAP can be approximatively considered as O(K×P×T×D), where *K* is the external number of iterations, not pulses in CPI (search for the collection of clutter atoms), *P* is the population size, *T* is the number of internal iterations (find the best atom via meta-heuristic algorithm), and *D* is the dimension of the data. With the same population size and number of internal iterations, the SI algorithms (PSO and GWO) generally require fewer external iterations than the EC algorithms (DE and GA) to reach the threshold, which means $K_{EC}>K_{SI}$. Hence, the time complexity of EC-STAP is higher than that of SI-STAP in general. The average runtimes of the five algorithms under the same threshold conditions are shown in the Table 2, which verifies the conclusions of the time complexity analysis.

In conclusion, compared with the traditional method, meta-heuristic algorithms can vastly improve the performance of SR-STAP with certain increases in the amount of calculation. PSO-STAP and GWO-STAP show better clutter suppression and less computational complexity, making them promising prospects for SR-STAP.

## 3. Improved PSO-GWO-STAP

The observations indicate that both the PSO and GWO algorithms demonstrate exceptional efficacy in SR-STAP. In general, PSO may converge on local optimal solutions, and GWO can maintain the balance between local and global search [30]. Therefore, PSO and GWO are combined in an iterative process to improve the overall performance. Concurrently, the fitness function is improved based on prior information in this section.

### 3.1. Improved Fitness Function

**Figure 5:**
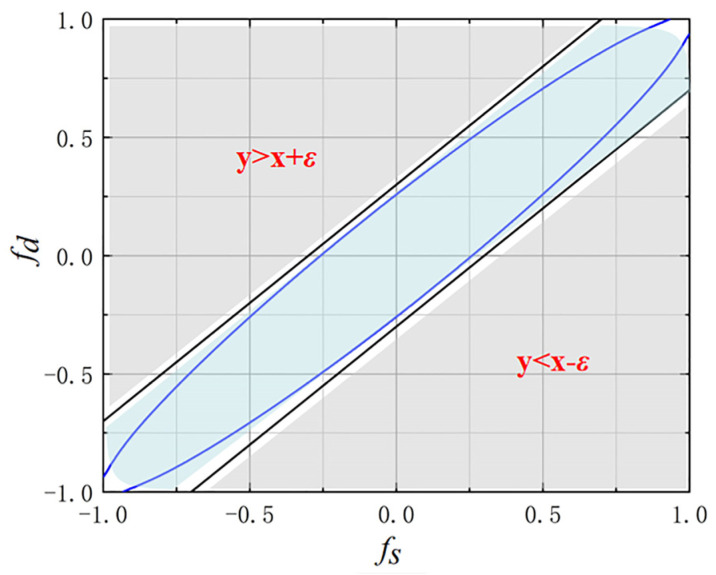
The diagram of the clutter distribution.

As shown in Figure 5, the spatial-Doppler plane can be divided into two parts based on the clutter distribution, which is determined by airborne platform and radar system parameters. Based on the above prior information, we propose a constraint factor *C* using the following formula:

(11) $$C=\left\{\begin{array}{cc} 1, & \mathrm{if}\;\left|f_{s}-f_{d}\right|<\varepsilon \\ e^{-\left|f_{s}-f_{d}\right|/2}, & \mathrm{else} \end{array}\right.$$

The expression for the new fitness function is as follows:

(12) $$F_{N}(a)=C\cdot F(a)=C\cdot \frac{\left|S{\left(f_{d},f_{s}\right)}^{H}P_{n}^{k}\hat{R}P_{n}^{k}S(f_{d},f_{s})\right|}{\left|S{\left(f_{d},f_{s}\right)}^{H}P_{n}^{k}S(f_{d},f_{s})\right|}$$

where ε depends on the clutter distribution. When *θ_p_* = 15°, ε is taken to 0.3. The main role of *C* is to reduce the possibility of selecting particles outside the clutter distribution region, so as to construct a more accurate clutter subspace and improve the convergence performance of the algorithm.

### 3.2. PSO and GWO Algorithms

In PSO, the formula for a particle to update its velocity and position is as follows:

(13) $$\begin{array}{l} v_{i}^{t+1}=\omega^{t}v_{i}^{t}+c_{1}r_{1}(Pbest_{i}^{t}-x_{i}^{t})+c_{2}r_{2}(Gbest^{t}-x_{i}^{t})\\ x_{i}^{t+1}=x_{i}^{t}+v_{i}^{t+1} \end{array}$$

where v is the velocity vector; ***x*** is the position of the particle; *t* is the iteration number; $c_{1}$ and $c_{2}$ are acceleration coefficients; and $r_{1}$ and $r_{2}$ ∈ [0, 1] are random values. Pbest and Gbest denote the best individual position and the best global position, respectively:

(14) $$\begin{array}{l} Pbest_{i}^{t+1}=\left\{\begin{array}{ll} Pbest_{i}^{t}, & \mathrm{if}\;F(x_{i}^{t+1})\leq F(Pbest_{i}^{t})\\ x_{i}^{t+1}, & \mathrm{if}\;F(x_{i}^{t+1})>F(Pbest_{i}^{t}) \end{array}\right.\\ Gbest^{t+1}=\arg\max F(Pbest_{i}^{t+1}),1\leq i\leq i_{\max} \end{array}$$

and ω generally adopts a linear decreasing weight strategy:

(15) $$\omega^{t}=\frac{\left(w_{\max}-w_{\min})(T-t\right)}{T}+w_{\min}$$

where *T* is the maximum number of iterations, and $w_{\max}$ and $w_{\min}$ are the setting parameters.

In GWO, the course of the hunting process is expressed as follows:

(16) $$\begin{array}{c} \vec{D_{\alpha }}=\left|{\vec{C}}_{1}\cdot {\vec{X}}_{\alpha }(t)-\vec{X(t)}\right|\\ \vec{D_{\beta }}=\left|\vec{C_{2}}\cdot \vec{X_{\beta }}(t)-\vec{X}(t)\right|\\ \vec{D_{\sigma }}=\left|\vec{C_{3}}\cdot \vec{X_{\sigma }}(t)-\vec{X}(t)\right| \end{array}$$

(17) $$\begin{array}{c} \vec{X_{1}}(t)=\left|\vec{X_{\alpha }}(t)-A_{1}\cdot \vec{D_{\alpha }}\right|\\ \vec{X_{2}}(t)=\left|\vec{X_{\beta }}(t)-A_{2}\cdot \vec{D_{\beta }}\right|\\ \vec{X_{3}}(t)=\left|\vec{X_{\sigma }}(t)-A_{3}\cdot \vec{D_{\sigma }}\right| \end{array}$$

(18) $$\vec{X}(t+1)=\frac{{\vec{X}}_{1}(t)+{\vec{X}}_{2}(t)+{\vec{X}}_{3}(t)}{3}$$

where $\vec{D_{\alpha }}$, $\vec{D_{\beta }}$, and $\vec{D_{\sigma }}$ are the distances between the α, β, and δ wolves and the prey. $\vec{X_{\alpha }}$, $\vec{X_{\beta }}$, and $\vec{X_{\sigma }}$ are the current positions of the α, β, and δ wolves. ${\vec{X}}_{1}$, ${\vec{X}}_{2}$, and ${\vec{X}}_{3}$ are the adjusted positions guided by the α, β, and δ wolves, respectively. *t* is the number of current iterations. $\vec{A}$ and $\vec{C}$ are determined coefficients, and their calculation formulas are as follows:

(19) $$\vec{A}=2\vec{a}\cdot {\vec{r}}_{1}-\vec{a}$$

(20) $$\vec{C}=2\cdot {\vec{r}}_{2}$$

where $\vec{r}$ and ${\vec{r}}_{2}$ are random numbers in [0, 1]; $\vec{a}$ linearly decreases from 2 to 0 as the number of iterations increases. The expression for $\vec{a}$ is as follows:

(21) $$\vec{a}=2-2\left(\frac{t}{T}\right)$$

where *T* is the maximum number of iterations.

### 3.3. The Process of the Proposed Method

As previously mentioned, the PSO algorithm has its own drawbacks. Therefore, PSO and GWO are combined to form a new optimizer to obtain better clutter suppression performance. The PSO-GWO algorithm process is as follows:

Step 1: Set the population size *P*, boundary conditions, and maximum number of iterations *T*, and randomly generate two populations (P1 for PSO and P2 for GWO). In other words, initialize parameters including the particle position $x_{i}^{0}$, velocity $v_{i}^{0}$, and grey wolf position $\vec{X}(0)$.

Step 2: The fitness value of each particle and wolf is calculate using (12). According to the results, the α wolf current position $\vec{X_{\alpha }}(0)$, β wolf current position $\vec{X_{\beta }}(0)$, and δ wolf current position $\vec{X_{\sigma }}(0)$ are obtained in the P2 population. Meanwhile, $Pbest_{i}^{0}$ and $Gbest^{0}$ are obtained in the P1 population.

Step 3: Update $\vec{X}(t+1)$ using (16)–(21), and update $\vec{X_{\alpha }}(t+1)$, $\vec{X_{\beta }}(t+1)$, and $\vec{X_{\sigma }}(t+1)$.

Step 4: Update $x_{i}^{t+1}$ and $v_{i}^{t+1}$ using (13) and (15).

Step 5: Sort $x_{i}^{t+1}$ by the fitness function (12) from largest to smallest, and update $x_{P}^{t+1}$=$\vec{X_{\alpha }}$,$x_{P-1}^{t+1}$=$\vec{X_{\beta }}$, and $x_{P-2}^{t+1}$=$\vec{X_{\sigma }}$.

Step 6: Update $Pbest_{i}^{t+1}$ and $Gbest^{t+1}$ using (14).

Step 7: Steps 3 to 6 are repeated until the number of iterations *t* reaches the set maximum number *T*, and the final Gbest is the optimal solution of the algorithm.

**Figure 6:**
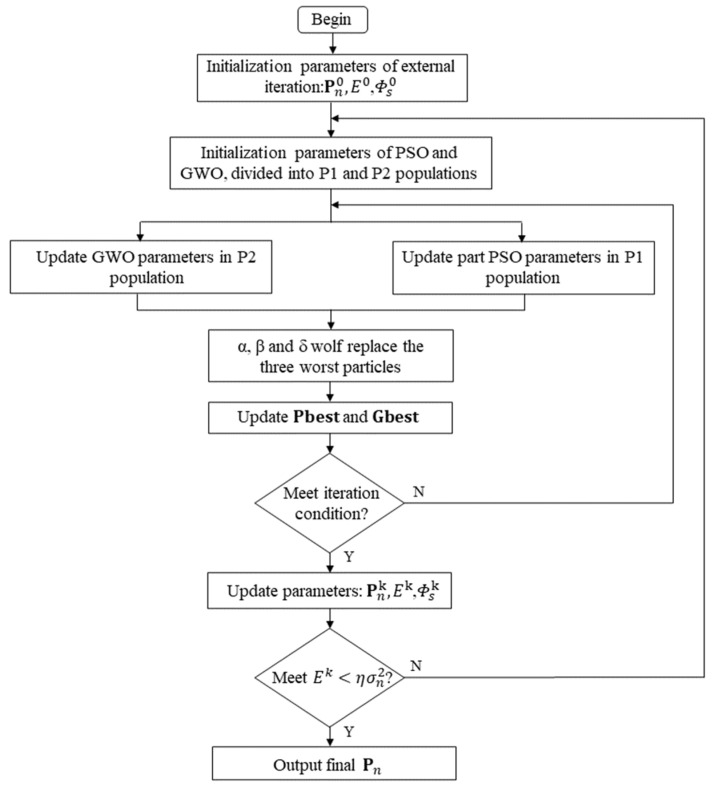
The process of the improved PSO-GWO-STAP.

The improved PSO-GWO-STAP is a method to obtain the optimal space-time steering vectors using the PSO-GWO algorithm; the process of the proposed model is shown in Figure 6.

### 3.4. The Performance of the Proposed Method

**Figure 7:**
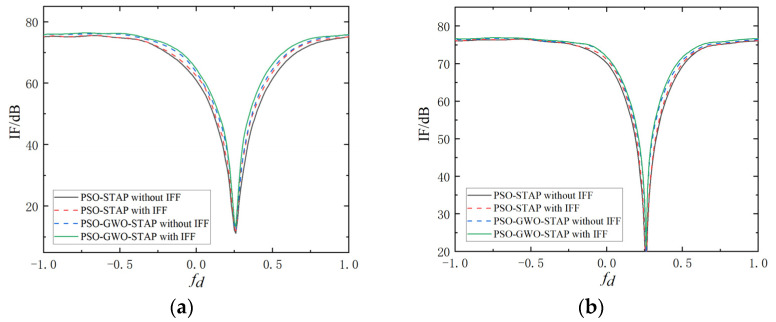
Comparison of IF curves. (**a**) *P* = 20, *T* = 5. (**b**) *P* = 30, *T* = 10.

This subsection first analyzes the impact of the improved fitness function and the PSO-GWO algorithm on clutter suppression performance in SR-STAP. The improvement factor (IF) is regarded as the main evaluation parameter, and the results are shown in Figure 7a,b with different population sizes (*P*) and iteration times (*T*).

It can be seen that compared with the single MH-STAP method, both the improved fitness function (IFF) and the PSO-GWO algorithm can improve the performance of SR-STAP. With the increase of the algorithm population size and the number of iterations, the performance improvement brought by the improved fitness function becomes limited, but the PSO-GWO algorithm continues to demonstrate its advantages. The reason for this phenomenon is that with the increase in computational complexity, the search capability of the algorithm is strengthened, allowing for better solutions without external constraints. Moreover, the most critical aspect of MH-STAP is to accurately find the clutter atom subset, which is limited by the search capability of the chosen meta-heuristic algorithm. A single meta-heuristic algorithm can improve its search capability by increasing the population size and iteration number, and it is highly likely to fall into local optima. The technique of algorithm hybridization can achieve global optimization under limited conditions. Therefore, PSO-GWO-STAP can obtain a more accurate clutter subspace than PSO-STAP, and its performance improvement is not affected by increases in population and iterations in this case.

The time complexity of the PSO-GWO-STAP algorithm can be considered as $O(K_{PSO-GWO}\times P_{sub-PSO}\times (T_{sub-PSO}+1)\times D_{sub-PSO})+O(K_{PSO-GWO}\times P_{sub-GWO}\times T_{sub-GWO}\times D_{sub-GWO})$; sub-PSO and sub-GWO represent the PSO part and GWO part of the PSO-GWO algorithm, respectively. Although $K_{PSO-GWO}<K_{PSO},K_{GWO}$ in general, all else being equal, PSO-GWO-STAP still has higher complexity than PSO-STAP (or GWO-STAP) in this case. In fact, the performance improvement brought about by increasing the population size and the number of iterations is also limited, so we set the sub-population size of PSO-GWO to 30, while the population size of the single PSO algorithm is set to 50, and the number of iterations is set to 50. Under such conditions, the performances of PSO-STAP and improved PSO-GWO-STAP have basically reached the optimal level. Therefore, this means that relatively fewer computational resources are consumed to improve the performance of the improved PSO-GWO-STAP method. The average runtime of PSO-GWO-STAP is 3.01 s, which is the same magnitude as single MH-STAP.

**Figure 8:**
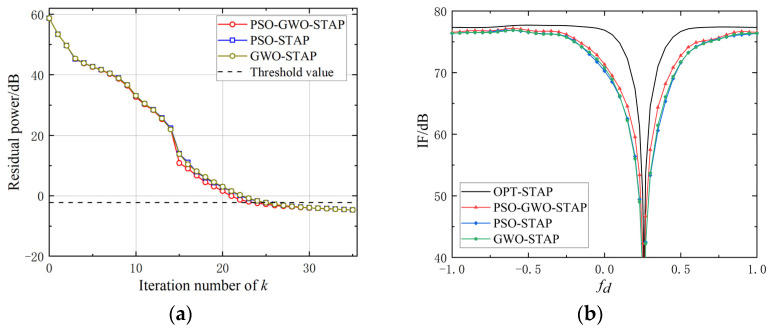
Comparison between PSO-STAP, GWO-STAP, and improved PSO-GWO-STAP. (**a**) Residual power. (**b**) IF curve.

As shown in Figure 8a, the residual power of the proposed PSO-GWO-STAP method decreased slightly faster than those of PSO-STAP and GWO-STAP, eventually aligning in later stages. Notably, it only takes 24 iterations for PSO-GWO-STAP to reach the threshold. In addition, it can be seen that the IF of the proposed PSO-GWO-STAP method is narrower in Figure 8b, which denotes superior performance in clutter suppression.

**Figure 9:**
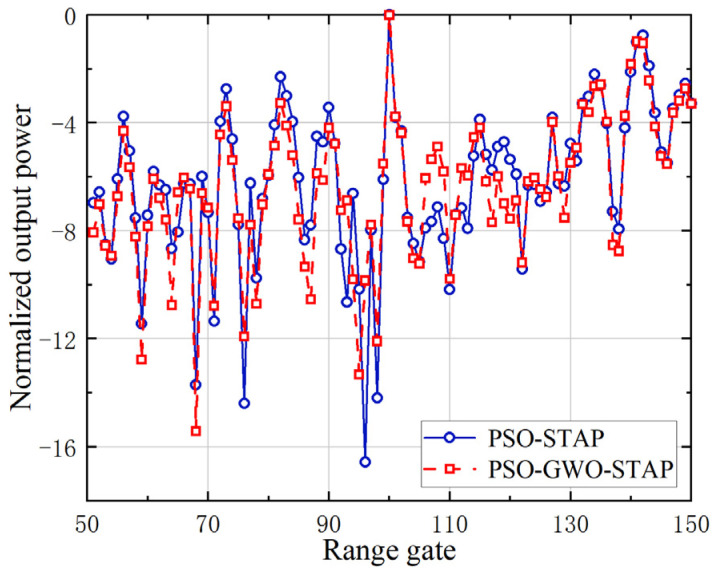
Normalized output power of improved PSO-GWO-STAP and PSO-STAP.

Furthermore, the actual target detection capability of the proposed PSO-GWO-STAP method is verified with mountain-top data. In Figure 9, a comparison of the normalized output power with the PSO-STAP method is provided, where the target was positioned at the 100th range gate. It can be observed that both algorithms can effectively detect a target which is located at this location. The normalized output power of PSO-GWO-STAP is slightly weaker than that of PSO-STAP, which means greater clutter suppression and target detection capabilities.

## 4. Conclusions

In this study, an improved PSO-GWO-STAP algorithm is proposed for airborne radar to solve the off-grid problem of SR-STAP. First, we apply meta-heuristic algorithms (GA, DE, PSO, and GWO) to SR-STAP to achieve meshless searching. Simulation and experimental results show that, compared with the traditional method, MH-STAP methods can more accurately find atoms on a spatial-Doppler profile. Furthermore, PSO-STAP and GWO-STAP show better clutter suppression performance than the other methods. Then, the fitness function is improved based on the prior information of the clutter distribution, which can improve the performance of MH-STAP under certain conditions. To obtain more excellent performance, PSO-GWO-STAP is created by combining GWO and PSO. GWO will transmit information to PSO in each iteration to improve the global optimization ability. The results show that the improved PSO-GWO-STAP algorithm can construct a more accurate clutter subspace than single MH-STAP. The limitation of the proposed method is that it consumes certain computing resources while improving the performance. 

In the future, we will research a low-computational-complexity algorithm which can achieve a greater improvement in clutter suppression performance for SR-STAP. Meanwhile, the proposed new fitness function can play a limited role only if it meets certain conditions, we will also research on a more universal function based prior information to further improve performance.

## Figures and Tables

**Table 1:** Simulation parameters.

Parameter	Symbol	Value
Platform height	*H*	8000 m
Signal wavelength	*λ*	0.23 m
Array spacing	*d*	0.115 m
Pulse repeat frequency	*f_r_*	2434.8 Hz
Flight velocity	*V*	140 m/s
Element number in UPA	*M × N*	8 × 8
Pulse number in CPI	*K*	8
Clutter-to-noise ratio	*CNR*	60 dB
Non-side-looking angle	*θ_p_*	15°

**Table 2:** Average runtimes of five algorithms.

STAP Algorithm	LMSSE	PSO	GWO	GA	DE
Average runtime(s)	1.53	1.93	1.97	2.14	7.64

## Data Availability

The data presented in this study are available on request from the corresponding author. The data are not publicly available due to restrictions related to privacy.

## Abbreviations

**Full Name**	**A** **cronym**
Clutter covariance matrix	CCM
Coherent processing interval	CPI
Differential evolution	DE
Direction of arrival	DOA
Deep unfolding	DU
Evolutionary computation	EC
Genetic algorithm	GA
Grey wolf optimization	GWO
Improvement factor	IF
Improved fitness function	IFF
Local mesh splitting subspace estimation	LMSSE
Meta-heuristic algorithms	MH
Moving target indication	MTI
Particle swarm optimization	PSO
Reduced-dimension local search clutter subspace estimation	RD-LSCSE
Sample covariance matrix	SCM
Swarm intelligence	SI
Sparse recovery	SR
Space-time adaptive processing	STAP
Uniform planar array	UPA
Wireless sensor networks	WSNs
